# Efficient human activity recognition with spatio-temporal spiking neural networks

**DOI:** 10.3389/fnins.2023.1233037

**Published:** 2023-09-14

**Authors:** Yuhang Li, Ruokai Yin, Youngeun Kim, Priyadarshini Panda

**Affiliations:** Department of Electrical Engineering, Yale University, New Haven, CT, United States

**Keywords:** brain-inspired computing, neuromorphic computing, human activity recognition, spiking neural networks, hardware efficiency

## Abstract

In this study, we explore Human Activity Recognition (HAR), a task that aims to predict individuals' daily activities utilizing time series data obtained from wearable sensors for health-related applications. Although recent research has predominantly employed end-to-end Artificial Neural Networks (ANNs) for feature extraction and classification in HAR, these approaches impose a substantial computational load on wearable devices and exhibit limitations in temporal feature extraction due to their activation functions. To address these challenges, we propose the application of Spiking Neural Networks (SNNs), an architecture inspired by the characteristics of biological neurons, to HAR tasks. SNNs accumulate input activation as presynaptic potential charges and generate a binary spike upon surpassing a predetermined threshold. This unique property facilitates spatio-temporal feature extraction and confers the advantage of low-power computation attributable to binary spikes. We conduct rigorous experiments on three distinct HAR datasets using SNNs, demonstrating that our approach attains competitive or superior performance relative to ANNs, while concurrently reducing energy consumption by up to 94%.

## 1. Introduction

In recent years, the proliferation of smart devices, such as smartphones and fitness trackers, has led to a growing interest in understanding user activities and behavior for healthcare applications. Human Activity Recognition (HAR) (Lara and Labrador, 2012; Anguita et al., 2013; Vrigkas et al., 2015) is an area of research that aims to identify user activities, with applications spanning sports injury detection, well-being management, medical diagnostics, smart building solutions (Ramanujam et al., 2021), and elderly care (Nweke et al., 2019). To accomplish these objectives, HAR tasks rely on specific input patterns derived from various sensors embedded in smart devices, including accelerometers, gyroscopes, and electroencephalogram (EEG) sensors. As the data collected from wearable sensors are time series in nature, the recognition of temporal patterns in sensor data is crucial for achieving high accuracy and efficiency.

Traditionally, researchers have employed hand-crafted features and straightforward classifiers for HAR tasks. Feature extraction techniques can be broadly categorized into statistical and structural (Figo et al., 2010; Bulling et al., 2014). Statistical features, such as mean, median, time domain, and frequency domain, encapsulate the distribution properties of individual training data samples. In contrast, structural methods account for the interactions between different training data samples, exemplified by techniques like principal component analysis (PCA), linear discriminant analysis (LDA), and empirical cumulative distribution functions (ECDF) (Abidine et al., 2018). Employing machine learning-based classifiers (Kim and Ling, 2009; Aggarwal and Xia, 2014; Shoaib et al., 2016) in conjunction with hand-crafted features has resulted in reasonably satisfactory performance.

**Figure 1:**
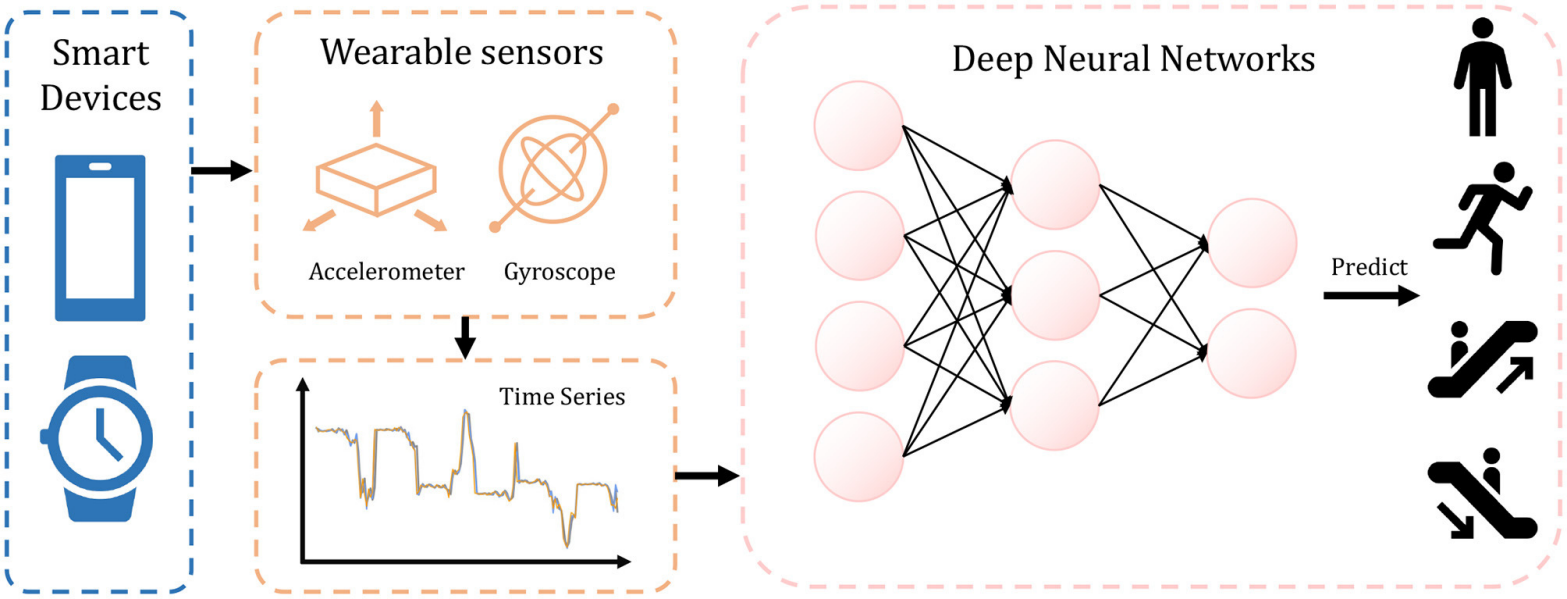
The overall HAR task procedure with ANN. Collected from smart devices, the sensor data are processed by ANN which recognizes user activity.

In more recent studies, deep learning techniques have been adopted for end-to-end feature extraction and classification in HAR tasks (Nweke et al., 2018). These approaches employ convolutional layers in Artificial Neural Networks (ANNs) (Mnih et al., 2015; Ignatov, 2018; Wan et al., 2020) and optimize the model using gradient backpropagation. Due to the capacity of gradient descent optimization to automatically determine the most suitable parameters, ANNs have demonstrated proficient performance across diverse datasets. Figure 1 illustrates the process of this algorithm, where the ANN utilizes time series data from wearable sensors to predict human activity.

However, we contend that ANNs, which employ full precision (32-bit) computation and exhibit low sparsity, impose considerable computational complexity and energy consumption on wearable devices. As expounded by Rastegari et al. (2016) and Qin et al. (2020), 32-bit networks necessitate 58 × more operations compared to fully 1-bit networks. Furthermore, ANNs rely on ReLU neurons (Krizhevsky et al., 2012) that do not account for temporal correlations. This design choice may be suboptimal, particularly for time series data, as it simply adapts the ANN framework from the image domain.

Due to the high-efficiency demands on wearable devices, reducing the memory and computation cost of HAR models has been a crucial research problem. As an example, Cheng et al. (2022) propose to use an ensemble of a set of experts, where each expert is a simple linear feature extractor. In addition, Tang et al. (2020) use Lego bricks as lower-dimension filters. Although these methods reduce the hardware cost to a certain extent, they lack direct optimization on the hardware side. As we mentioned, an extremely low-bit neural network can reduce hardware costs by an order of magnitude (Li et al., 2019).

Hypothetically, simply adapting the current architecture to 1-bit networks will greatly impact the representation ability, and thus reduce the accuracy of HAR. To address this problem, one has to consider how to extract the temporal information in sensor data more effectively with discrete and limited 1-bit representation.

To address the aforementioned problems, we employ Spiking Neural Networks (SNNs) (Roy et al., 2019; Tavanaei et al., 2019; Deng et al., 2020; Panda et al., 2020; Li et al., 2021b; Xu et al., 2022, 2023; Zhu et al., 2022) in conjunction with convolutional layers for processing time series data in HAR tasks. HAR can benefit from SNNs in two key aspects: (1) SNNs leverage binary spikes (either 0 or 1) for activation, enabling multiplication-free and highly sparse computation, thereby reducing energy consumption for time series data (Zhang et al., 2018, 2021; Wu et al., 2021); (2) SNNs inherently model the temporal dynamics present in time series data, as spiking neurons within SNNs maintain a variable called the membrane potential over time. When the membrane potential surpasses a predefined threshold, the neuron fires a spike in the current time step. Capitalizing on these two advantages, our SNNs exhibit comparable or even superior performance to ANNs.

Additionally, we extend a previous hardware accelerator design to support 1D convolution along the time dimension, making it suitable for SNN implementation (Yin et al., 2022). We evaluate our SNNs on three widely-used HAR datasets (UCI-HAR Anguita et al., 2013, UniMB SHAR Micucci et al., 2017, HHAR Stisen et al., 2015) and compare them with ANN baselines. Our SNNs achieve the same or higher accuracy than ANNs while reducing energy consumption by up to 94%.

In summary, our contributions are 3-fold:

1. We propose the use of SNNs for HAR tasks, significantly reducing energy consumption while integrating a temporally-evolving activation function.
2. We design a hardware accelerator tailored for deploying SNNs on edge devices.
3. We conduct extensive experiments on three HAR benchmarks, demonstrating that our SNNs outperform ANNs in terms of accuracy while maintaining energy-saving advantages.

## 2. Materials and methods

### 2.1. Notations

We use bold lower letters for vector representations. For example, *x* and *y* denote the input data and target label variables. Bold capital letters like **W** denote the matrices (or tensors as clear from the text). Constants are denoted by small upright letters, e.g., *a*. With bracketed superscript and subscript, we can denote the time dimension and the element indices, respectively. For example, $x_{i}^{\left(t\right)}$ means the *i*-th training sample at time step *t*.

### 2.2. Background of HAR

Concretely, we denote the wearable-based sensor dataset with ${\left\{x_{i}\right\}}_{i=1}^{N}$, and each sample ***x**i* ∈ ℝ^T×D^ is collected when the wearer is doing certain activity **y**_*i*_, e.g., running, sitting, lying, standing, etc. Here, data samples are streaming and have *T* time steps in total. *D* is the dimension of the sensor's output. As an example, the accelerometer records the acceleration in the (*x, y, z*)-axis, thus *D* = 3 for the accelerometer data. We are interested in designing an end-to-end model *f*(·) and optimizing it to predict the activity label **y**.

### 2.3. Spiking neuron

In this section, we introduce the definition of spiking neurons. We adopt the well-known Leaky-Integrate-and-Fire (LIF) neuronal model for spiking neurons (Liu and Wang, 2001), which constantly receives input and fires spikes through time. Formally, the LIF neuron maintains the membrane potential *v* through time, and at *t*-th time step (1 ≤ *t* ≤ *T*), the membrane potential receives the pre-synaptic input charge *c*^(*t*)^, given by


(1) $$\begin{array}{l} v^{\left(t+1\right),\text{pre}}=\tau v^{\left(t\right)}+c^{\left(t\right)},\text{where }c^{\left(t\right)}=Ws^{\left(t\right)}. \end{array}$$

Here, τ is a constant between [0, 1] representing the decay factor of the membrane potential as time flows, which controls the correlation between time steps. τ = 0 stands for 0 correlation and LIF degenerates to binary activation (Rastegari et al., 2016) without temporal dynamics, while τ = 1 stands for maximum correlation and Deng and Gu (2021) and Li et al. (2021a) proves that LIF will become ReLU neuron when *T* is sufficiently large. *c*^(*t*+1)^ is the product between weights **W** and the spike *s*^(*t*+1)^ from previous layer. After receiving the input charge, the LIF neuron will fire a spike if the pre-synaptic membrane potential exceeds some threshold, given by


(2) $$\begin{array}{l} s^{\left(t+1\right)}=\{\begin{array}{cc} 1 & \text{if }v^{(t+1),\text{pre}}>V_{th}\\ 0 & \text{otherwise} \end{array}, \end{array}$$

where *V*_*th*_ is the firing threshold. Note that the spike *s*^(*t*+1)^ will propagate to the next layer, here we omit the layer index for simplicity.

**Figure 2:**
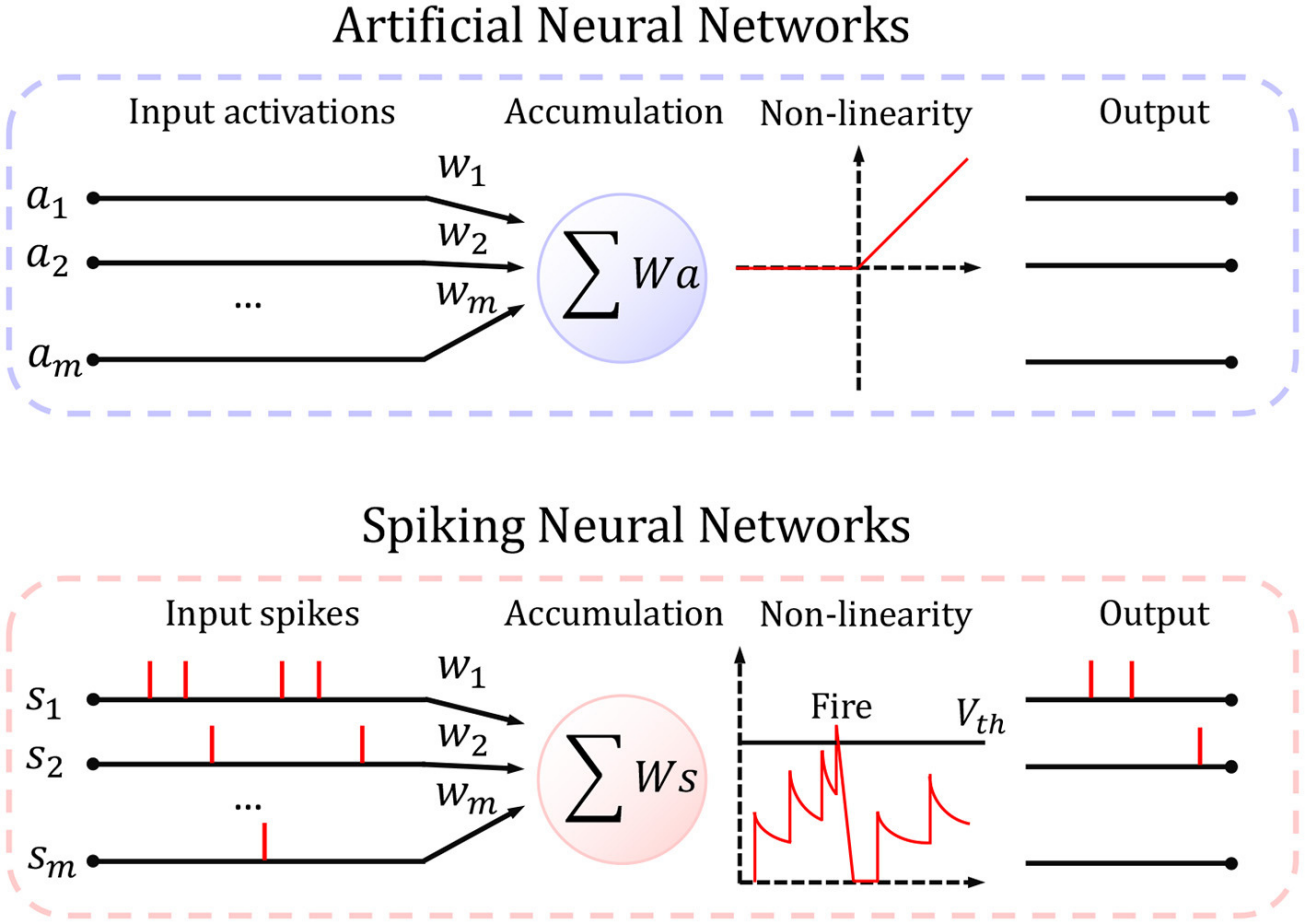
The schematic view of artificial neurons and spiking neurons. Artificial neuron takes full precision input and rectifies it if it is < 0 and pass it otherwise; spiking neuron considers the correlation between times, and fire a spike only if the membrane potential is higher than a threshold.

If the LIF neurons fire a spike, the membrane potential will be reset. This can be done by either soft-reset or hard-reset, denoted by


(3) $$\begin{array}{l} \{\begin{array}{cc} v^{\left(t+1\right)}=v^{(t+1),\text{pre}}\;\cdot \;(1-s^{\left(t+1\right)}) & \text{\# Hard-Reset}\\ v^{\left(t+1\right)}=v^{(t+1),\text{pre}}-s^{\left(t+1\right)}\;\cdot \;V_{th} & \text{\# Soft-Reset} \end{array}, \end{array}$$

where hard-reset sets *v*^(*t*+1)^ to 0, while soft-reset subtracts *v*^(*t*+1)^ by *V*_*th*_. We choose LIF neurons because *s*^(*t*+1)^ is binary and dependent on input in previous time steps. Figure 2 describes the difference between ANN and SNN in a systematic way. In our experiments, we will conduct ablation studies on the decay factor, the firing threshold, and the reset mechanism.

#### 2.3.1. Integrating spiking neurons into ANN

We first integrate spiking neurons into artificial neural networks by replacing their non-linear activation with LIF. As a result, we can compare the performance between artificial neurons and spiking neurons. Specifically, since the time series data naturally has a time dimension, we also integrate the pre-synaptic potential charge along this time dimension. For instance, suppose *a*∈ℝ^*n*×*c*×*T*^ is a pre-activation tensor, where *n, c, T* represent the batch size, channel number, and total time steps, respectively. We set the charge in each time step for LIF as the pre-activation in the corresponding time step, i.e., $c^{\left(t\right)}=a_{:,:,t}$. Then, we stack the output spikes along the time dimension again, i.e., $\text{S}=\text{stack}\left({\left\{s^{\left(t\right)}\right\}}_{t=1}^{T}\right)$, for calculating the pre-activation in next layer.

### 2.4. Optimization

Although LIF neurons manage to model the temporal features and produce binary spikes, the firing function (Equation 2) is discrete and thus produces zero gradients almost everywhere, prohibiting gradient-based optimization. Particularly, the gradient of loss (denoted by *L*) w.r.t. weights can be computed using the chain rule:


(4) $$\begin{array}{l} \begin{array}{l} \;\frac{\partial L}{\;\partial W}=\sum_{t=1}^{T}\frac{\partial L}{\partial s^{\left(t\right)}}\frac{\partial s^{\left(t\right)}}{\partial v^{(t),\text{pre}}} \end{array}\\ \left(\frac{\partial v^{(t),\text{pre}}}{\partial c^{\left(t\right)}}\frac{\partial c^{\left(t\right)}}{\partial W}+\sum_{t^{\prime }=1}^{t-1}\frac{\partial v^{(t),\text{pre}}}{\partial v^{\left(t^{\prime }\right)}}\frac{\partial v^{\left(t^{\prime }\right)}}{\partial v^{(t^{\prime }),\text{pre}}}\frac{\partial v^{(t^{\prime }),\text{pre}}}{\partial c^{\left(t^{\prime }\right)}}\frac{\partial c^{\left(t^{\prime }\right)}}{\partial W}\right). \end{array}$$

Here, all terms can be differentiated except $\frac{\partial s^{\left(t\right)}}{\partial v^{\left(t\right),\text{pre}}}$ which brings zero-but-all gradients. To circumvent this problem, we use the surrogate gradient method. In detail, we use the triangle surrogate gradient, given by


(5) $$\begin{array}{l} \frac{\partial s^{\left(t\right)}}{\partial v^{(t),\text{pre}}}=\max\left(0,1-\left|\frac{v^{(t),\text{pre}}}{V_{th}}-1\right|\right). \end{array}$$

**Figure 3:**
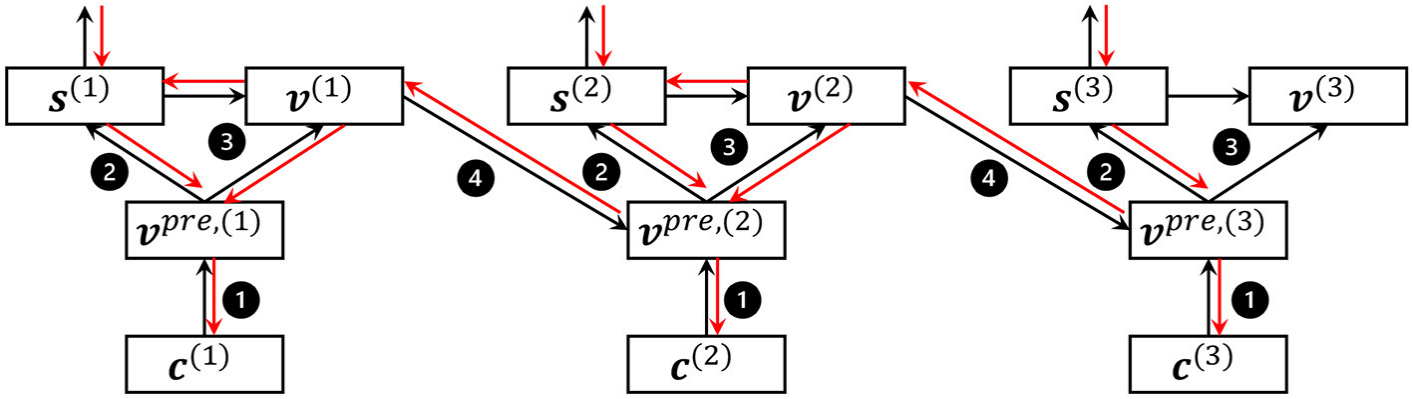
An example of the forward and backward process of LIF neurons in 3 time steps. → , forward; →, backward; ❶, potential charge; ❷, fire; ❸, reset; ❹, integrate and decay.

As a result, SNNs can be optimized with stochastic gradient descent algorithms, as shown in Figure 3.

### 2.5. Hardware implementation

Finally, we introduce the hardware platform that we design for carrying out the experiments on energy efficiency. We extend the overall architecture and PE design from Yin et al. (2022) to support the necessary computation and data movement for our SNNs in HAR tasks. Owing to the 1D convolution and temporal dynamics that are naturally embedded in the time series data, the complexity of the hardware design has been largely reduced.

**Figure 4:**
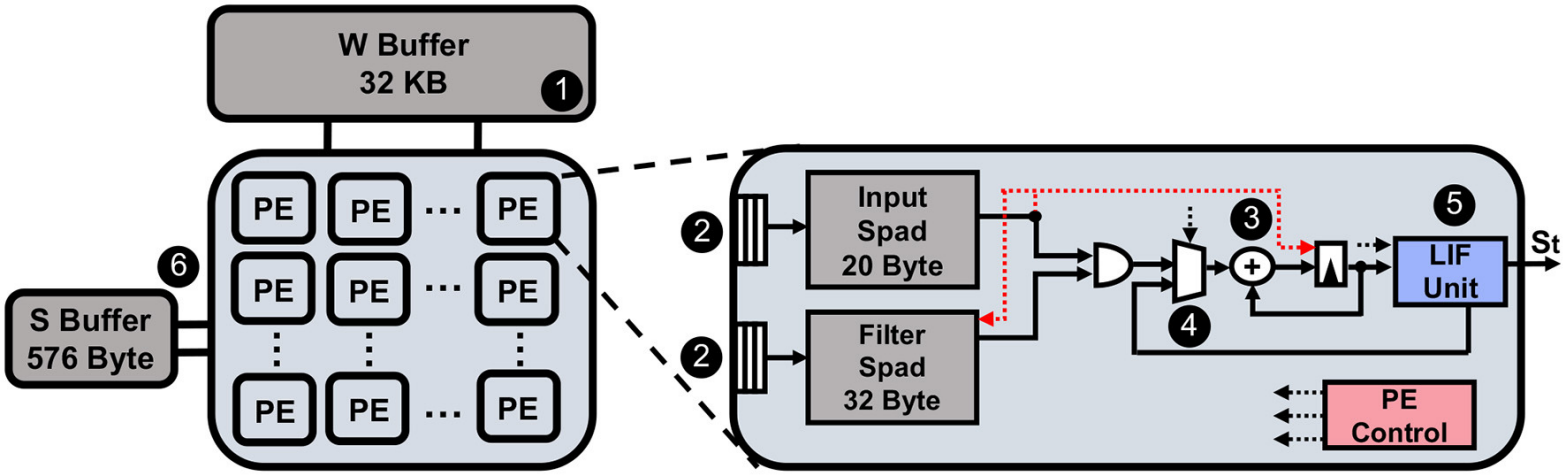
The illustration of the hardware design we used for the experiment.

As shown in Figure 4, our systolic-array-based hardware platform equips one PE array and two global buffers for holding the weights and spikes. The size of the PE array and global buffers are configurable according to different network structures. In this work, we set the number of PEs to 128, weight (W) buffer to 32 KB, and spike (S) buffer to 576 bytes, for matching with the dataflow used in Yin et al. (2022). We briefly explain the computation and data movement flow below.

In Figure 4, at step ❶, the entire weights for the layer are fetched into the global buffer from DRAM. The weights and the spikes will be written into the scratchpads inside PEs at step ❷. At step ❸, the accumulation is carried out for computing the *Ws*^(*t*)^ and the partial sum result is added with the residual membrane potential from time step *t*−1 at step ❹. The latest membrane potential for time step *t* is then sent to the LIF unit at step ❺ to generate the output spike *s*^(*t*)^. According to the dataflow in Yin et al. (2022), each PE will only focus on working on one output neuron, and the PE array processes the whole output feature map in parallel. After that, spikes for the next layer will be written into the S buffer at step ❻, and the whole process will repeat. Note that we can directly apply the input spike to skip the accumulation and weight scratchpad access if the input is equal to zero. We show the energy cost for each operation on the PE level in Table 1 for the reader's reference. Here, *E*_*mac*_ is the energy cost for a single multiply-accumulate (MAC) operation (note that the multiplication with spikes becomes logical AND operation between spikes and weights); *E*_*spa*_ is the energy cost for handling spike sparsity; *E*_*LIF*_ is the energy of a LIF operation; *E*_*Ispad*_ and *E*_*Wspad*_ are the single access energy to the input and weight scratchpad separately. All of the values are normalized by the energy cost of a MAC operation in ANN.

**Table 1:** Normalized energy cost for each operation on the PE level.

**Operation**	**Normalized energy cost**
*E*_*mac*_	0.175
*E*_*spa*_	0
*E*_*LIF*_	0.383
*E*_*Ispad*_	0.107
*E*_*Wspad*_	1.712

The energy is normalized with the energy cost for one MAC operation in the ANN.

## 3. Experiments

In this section, we verify the effectiveness and efficiency of our SNNs on three popular HAR benchmarks. We first briefly provide the implementation details of our experiments and then compare our method with ANN baselines. Finally, we conduct ablation studies to validate our design choices.

### 3.1. Implementation details

We implement our SNNs and existing ANNs with the PyTorch framework (Paszke et al., 2019). For all our experiments, we use Adam optimizer (Kingma and Ba, 2014). All models are trained for 60 epochs, with batch size 128. The only flexible hyper-parameter is the learning rate, which is selected from {1*e*−4, 3*e*−4, 1*e*−3} with the best validation accuracy. We use Cosine Annealing Decay for the learning rate schedule. For all three HAR datasets, we split them to 64% as the training set, 16% as the validation set, and 20% as the test set. We report test accuracy when the model reaches the best validation accuracy. Note that these datasets only have one label for each input sample, therefore top-1 accuracy is the same as the F-1 score. Similar to the SNN in image recognition tasks (Kim et al., 2022), the last layer of our SNN architecture is a fully connected layer. Therefore, we simply integrate all the membrane potentials in this layer for the softmax class prediction. We use the vanilla cross-entropy loss function rather than other specific loss functions (Deng et al., 2022; Zhu et al., 2022) to optimize our model. The dataset descriptions are shown below:

UCI-HAR (Anguita et al., 2013) contains 10.3 k instances collected from 30 subjects. It involves 6 different activities including walking, walking upstairs, walking downstairs, sitting, standing, and lying. The sensors are the 3-axis accelerometer and 3-axis gyroscope (both are 50 Hz) from Samsung Galaxy SII.

UniMB SHAR (Micucci et al., 2017) contains 11.7 k instances collected from 30 subjects. It involves 17 different activities including 9 kinds of daily living activities and 6 kinds of fall activities. The sensor is the 3-axis accelerometer (maximum 50 Hz) from Samsung Galaxy Nexus I9250.

HHAR (Stisen et al., 2015) contains 57 k instances collected from 9 subjects. It involves 6 daily activities including biking, sitting, standing, walking, stair up, and stair down. The sensors are accelerometers from 8 smartphones and 4 smart watches (sampling rate from 50 to 200 Hz).

### 3.2. Comparison with ANNs

For ANN baselines, we select Convolutional Neural Networks (CNN) (Avilés-Cruz et al., 2019), DeepConvLSTM (DCL) (Mukherjee et al., 2020), Long Short Term Memory (LSTM) (Wang and Liu, 2020), and Transformer (Vaswani et al., 2017) architectures. We replace the ReLU neurons with spiking neurons, therefore, we can only integrate them into CNN and DeepConvLSTM since LSTM and Transformer have other activations like tanh and swish. The CNN architecture is marked by *C32-MP2-C64-MP2-C64-MP2-FC*, where each convolutional layer is a 1-dimensional convolution with a kernel size of 8. For DCL architecture, it is marked by *C64-C64-C64-C64-LSTM64, where each convolutional layer uses a kernel size of 5*. Dropout is applied in Artificial CNN and DCL to reduce redundant activations, but not in spiking CNN and DCL. Each result is averaged from 5 runs (random seeds from 1,000 to 1,004) and includes a standard deviation value.

We summarize the results in Table 2, from which we find that SNNs have higher accuracy than the ANNs. For example, on the UniMB SHAR dataset, SpikeCNN has a 1.7% average accuracy improvement over its artificial CNN counterpart. Even more remarkably, the SpikeDeepConvLSTM (SpikeDCL) on the UCI-HAR dataset reaches 98.86% accuracy, which is 1% higher than DCL. Considering the accuracy is approaching 100%, the 1% improvement would be very significant. For UCI-HAR and HHAR datasets, we find SpikeCNN has similar accuracy to CNN, instead, the SpikeDeepConvLSTM consistently outperforms DeepConvLSTM, indicating that SNNs can be more coherent with the LSTM layer. Regarding the standard deviation of accuracy, we find that SNNs are usually more stable than ANNs, except for only one case, SpikeCNN on UCI-HAR.

**Table 2:** Accuracy (%) comparison between different networks on three HAR datasets (DCL, DeepConvLSTM).

**Model**	**UCI-HAR (Anguita et al., 2013)**	**SHAR (Micucci et al., 2017)**	**HHAR (Stisen et al., 2015)**
CNN	96.29 ± 0.12	92.38 ± 0.51	96.19 ± 0.14
DCL	97.87 ± 0.32	90.78 ± 1.05	97.15 ± 0.17
LSTM	82.41 ± 4.04	83.87 ± 0.96	95.59 ± 0.20
Transformer	96.02 ± 0.27	83.19 ± 0.74	95.82 ± 0.16
SpikeCNN	**96.40** ±**0.15**	**94.04** ±**0.34**	**96.20** ±**0.09**
SpikeDCL	**98.86** ±**0.28**	**92.08** ±**0.77**	**97.52** ±**0.10**

The bold values indicate that they achieve the highest accuracy under the same architecture family.

We also compare our SNN with existing methods using ANNs on three HAR datasets. The results are summarized in Table 3. It can be found that our method achieves higher accuracy compared to these baselines, demonstrating the effectiveness of our method. For instance, our SpikeCNN has 1.7% higher accuracy than the CNN used in Ronao and Cho (2016) and our SpikeDCL obtains 1.5% higher accuracy than the DCL proposed in Zhu et al. (2018).

**Table 3:** Accuracy (%) comparison between our SNNs with existing ANNs, including CNN, LSTM, and DeepConvLSTM.

**Method**	**Model**	**UCI-HAR**	**SHAR**	**HHAR**
Ronao and Cho (2016)	CNN	94.79	-	-
Khan et al. (2018)	CNN	-	-	78.75
Wang and Liu (2020)	LSTM	91.65	-	85.82
Mukherjee et al. (2020)	DCL	-	**92.30**	-
Zhu et al. (2018)	DCL	97.31	-	-
Ours	SpikeCNN	**96.40** ±**0.15**	**94.04** ±**0.34**	**96.20** ±**0.09**
Ours	SpikeDCL	**98.86** ±**0.28**	92.08 ± 0.77	**97.52** ±**0.10**

The bold values indicate that they achieve the highest accuracy under the same architecture family.

### 3.3. Ablation studies

In this section, we conduct ablation studies with respect to the (hyper)-parameters in the LIF neurons, including decay factor, threshold, and reset mechanism. We test SpikeDCL and SpikeCNN on UCI-HAR and SHAR datasets.

#### 3.3.1. The effect of decay factor

We select 5 fixed decay factors from {0.0, 0.25, 0.5, 0.75, 1.0}. Note that as discussed before τ = 0 indicates no correlation between two consecutive time steps, therefore SNN becomes equivalent to Binary Activation Networks (BAN), while τ = 1 indicates full correlation. Additionally, we add another choice *parameterized* τ where the decay factor can be learned for each layer. This choice avoids the manual adjustments of the decay factor. Specifically, we initialize *b* = 0 and use τ = sigmoid(*b*) to represent the decay factor. The gradient w.r.t. *c* is given by


(6) $$\begin{array}{l} \begin{array}{l} \;\frac{\partial L}{\partial b}=\sum_{t=1}^{T}\frac{\partial L}{\partial s^{\left(t\right)}}\frac{\partial s^{\left(t\right)}}{\partial v^{(t),pre}} \end{array}\\ \begin{array}{l} \left(\frac{\partial v^{(t),pre}}{\partial \tau }\frac{\partial \tau }{\partial b}+\sum_{t^{\prime }=1}^{t-1}\frac{\partial v^{(t),pre}}{\partial v^{\left(t^{\prime }\right)}}\frac{\partial v^{\left(t^{\prime }\right)}}{\partial v^{(t^{\prime }),pre}}\frac{\partial v^{(t^{\prime }),pre}}{\partial \tau }\frac{\partial \tau }{\partial b}\right). \end{array} \end{array}$$

We provide all results in Table 4. We can find that τ has a huge impact on the final test accuracy. For the UCI-HAR dataset with SpikeDCL, the accuracy of τ = 0 is 94.36% while the accuracy of τ = 0.75 is 98.86%. Additionally, if we compare other 0 < τ < 1 cases with τ = 0, we find that τ = 0 always produces a large deficiency. *This indicates that considering the temporal correlation with* τ>0 *is necessary for the time series tasks*. It also verifies our hypothesis in Section 1 that simply using 1-bit without considering temporal information will degrade the accuracy. Moreover, for the SHAR dataset, the τ = 1 case only has 60.55 accuracy while the τ = 0.25 case achieves 91.72% accuracy.

**Table 4:** Ablation study on the decay factor τ.

**Dataset**	**Model**	**Decay factor** τ					

		**0.0**	**0.25**	**0.5**	**0.75**	**1.0**	**Param**
UCI-HAR (Anguita et al., 2013)	SpikeCNN	95.48	95.63	95.78	**96.40**	95.92	96.11
	SpikeDCL	94.36	96.50	97.57	**98.86**	96.60	97.37
SHAR (Micucci et al., 2017)	SpikeCNN	93.54	**94.04**	93.48	93.85	74.68	93.54
	SpikeDCL	89.53	**92.08**	90.93	90.10	60.55	91.53

The bold values indicate that they achieve the highest accuracy under the same architecture family.

It is also worthwhile to note that different datasets have varying optimal decay factor rates. The UCI-HAR favors 0.75 as its decay factor while the SHAR prefers 0.25. We think the primary reason for this change is that SHAR has sharper variation in its input and has a much larger range than UCI-HAR. Therefore, it should maintain a relatively low τ.

As for the parameterized decay factor, we do not observe its superiority over the fixed decay factor model. The parameterized τ generally achieves decent performance but not the best.

#### 3.3.2. The effect of firing threshold

We next study the effect of the firing threshold. Generally, the firing threshold is related to the easiness of firing a spike. We set the threshold as {0.25, 0.5, 0.75, 1.0} and run the same experiments with the former ablation. Here, through Table 5 we observe that the firing threshold has a unified pattern. SNN reaches its highest performance when the firing threshold is set to 0.5. This result is not surprising since 0.5 is in the mid of 0 and 1, and thus has the lowest error for the sign function. Meanwhile, we find the difference in accuracy brought by the firing threshold is lower than the decay factor. For instance, the largest gap when changing the threshold for SpikeDCL on the SHAR dataset is 0.65%, while this gap can be 32% when changing the decay factor. Therefore, the SNN is more sensitive to the decay factor rather than the threshold.

**Table 5:** Ablation study on the firing threshold *V*_*th*_ and the reset mechanism.

**Dataset**	**Model**	**Firing threshold** ***V***_*****th*****_				**Reset**	

		**0.25**	**0.5**	**0.75**	**1.0**	**Hard**	**Soft**
UCI-HAR (Anguita et al., 2013)	SpikeCNN	95.71	**96.40**	96.18	96.11	96.09	**96.40**
	SpikeDCL	98.27	**98.86**	97.60	96.81	98.53	**98.73**
SHAR (Micucci et al., 2017)	SpikeCNN	93.91	**94.04**	93.89	93.87	92.75	**94.04**
	SpikeDCL	91.42	**92.08**	91.72	91.53	91.13	**92.08**

The bold values indicate that they achieve the highest accuracy under the same architecture family.

#### 3.3.3. The effect of reset mechanism

Finally, we verify the reset mechanism for SNNs, namely soft-reset and hard-reset. The results are sorted in Table 5. For all cases, the soft-reset mechanism is better than the hard-reset. We think the reason behind this is that the hard reset will directly set the membrane potential to 0, therefore cutting off the correlation between intermediate time steps. Instead, the soft-reset keeps some previous time step's information on membrane potential after firing.

### 3.4. Hardware performance evaluation

**Figure 5:**
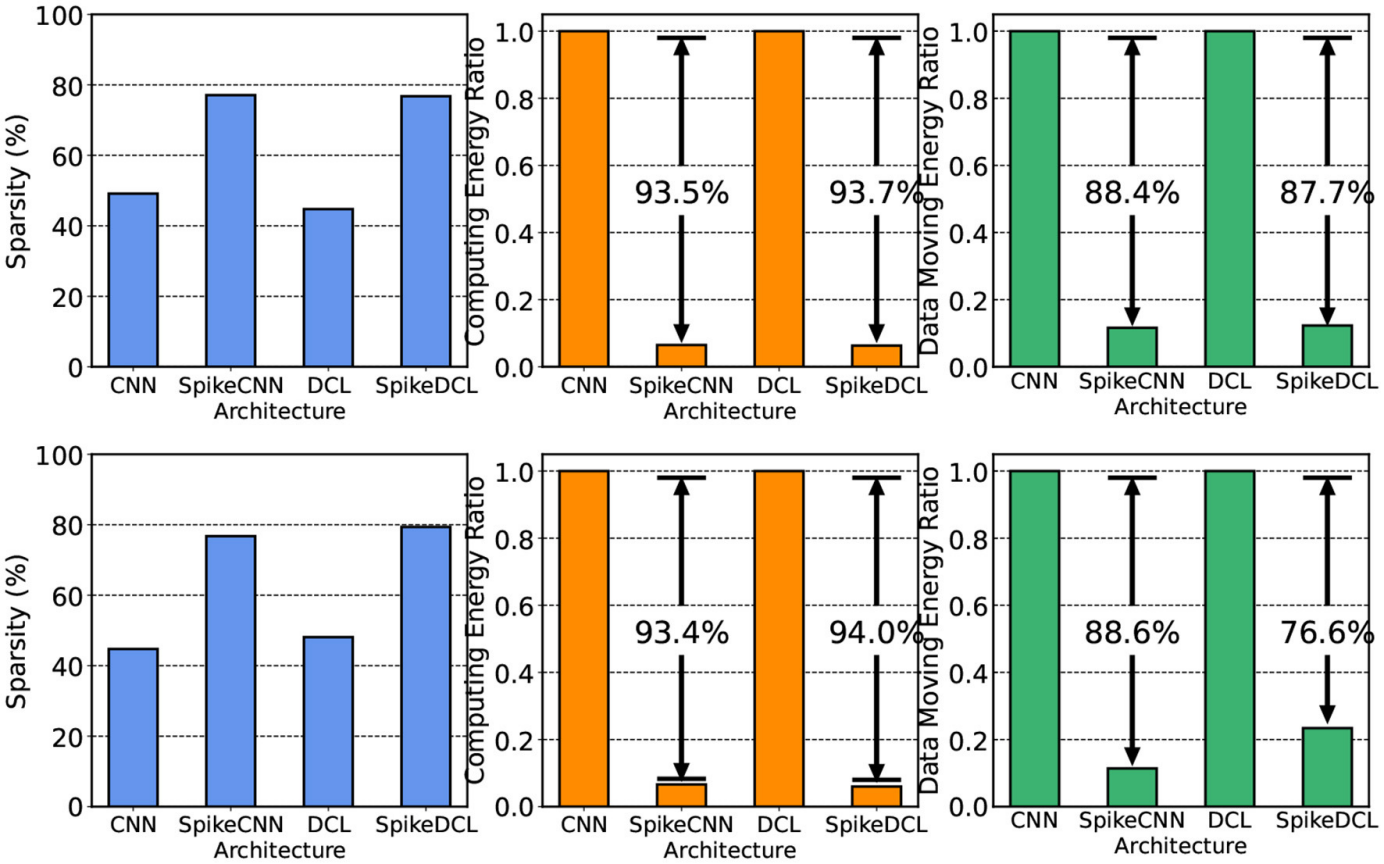
Hardware costs comparison between ANNs and SNNs on UCI-HAR and SHAR datasets, respectively. We include sparsity and energy consumption.

In this section, we compare the hardware performance between SNN and ANN. Here, we compare two metrics, namely the activation sparsity and the energy consumption. Higher sparsity can avoid more computations with weights in hardware that supports sparse computation. We measure the sparsity either in ReLU (ANNs) or in LIF (SNNs) and visualize them in Figure 5 (blue chart). The ReLU in ANN usually has around 50% sparsity, an intuitive result since the mean of activation is around 0. LIF neurons, however, exhibit a higher sparsity, approximately 80%, probably due to the threshold for firing being larger than 0. As a result, SNNs can save more operations in inference.

The second metric in hardware performance is energy consumption. We estimate the energy consumption by simulating the proposed hardware design in Section 2.5 together with our ReLU-based ANN baseline through the energy simulator proposed in Yin et al. (2022). The overall energy we consider consists of two parts: computing energy and data-moving energy. SNNs have advantages in computing energy due to their binary activation and higher sparsity. The results are shown in Figure 5 right. It can be seen that SNNs consume up to 94% less energy than ANNs, which could largely promote the battery life in smart devices. However, in the image processing domain, SNNs may have higher data-moving energy because they need to store the membrane potential and access them in the future (Yin et al., 2022, 2023; Moitra et al., 2023). We demonstrate that, in the HAR domain, SNNs have even lower data-moving energy than ANNs. The input data in HAR are augmented multiple times to generate the features in the time dimension. However, the SNNs in HAR do not need to increase the dimension of intermediate features to accommodate the time dimension resulting in lower data-moving costs. In summary, SNNs bring higher task performance due to the LIF neurons, and also energy efficiency due to the binary representation with high sparsity.

### 3.5. Convergence

**Figure 6:**
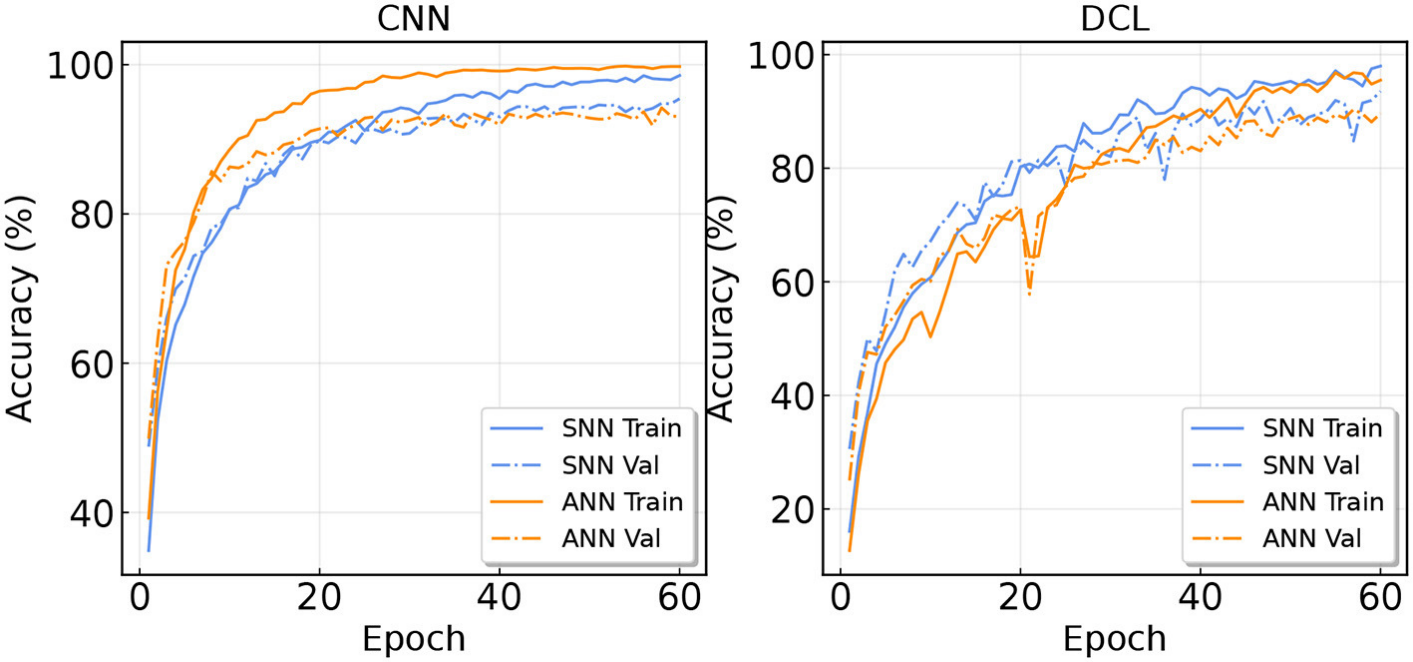
Training and validation curve on the SHAR dataset.

In this section, we visualize the convergence curves of both ANN and SNN. We record the training accuracy and validation accuracy during training for the CNN and DCL models. The curves are shown in Figure 6. In the first figure, we can find that the CNN converges faster than the SpikeCNN. The training accuracy of ANN is always higher than the SNN. The validation accuracy of ANN also maintains its advantages at first, however, the validation accuracy of SNN becomes higher in the later stages. We conjecture that in the pure convolutional architecture, SNN is harder to be optimized than ANN and it may have a smaller generalization gap due to its binary activation.

For the right side of Figure 5, we record the curves of DeepConvLSTM. It can be seen that SNN has faster convergence in this case. The validation accuracy of SNN is always higher than ANN. This result confirms that SNN is more coherent with LSTM layers.

### 3.6. Representation similarity

**Figure 7:**
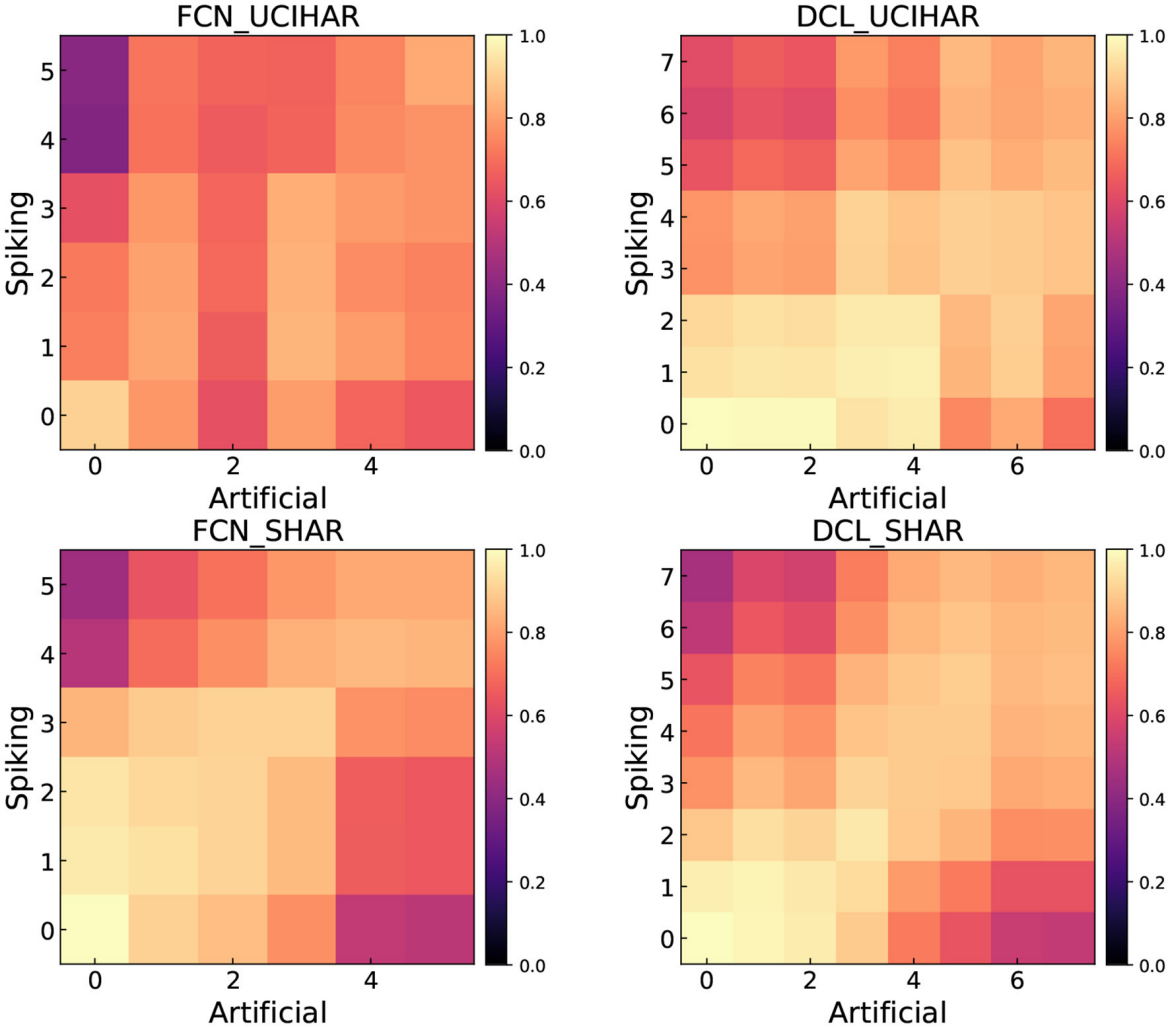
Feature similarity measure between ANN and SNN using CKA.

In this section, we visualize the similarity between the ANN's and SNN's representation. We use Centered Kernel Alignment (CKA) (Kornblith et al., 2019; Li et al., 2023) to calculate the representation similarity index. We compare CNN and DCL on UCI-HAR and SHAR datasets. We compute the CKA value between convolutional or activation layers, for ReLU and LIF. Therefore, we can construct a heatmap with *x, y* axes being the layer index, and each entry is the CKA value of layers with those indices. The heatmaps are shown in Figure 7. In general, we find that the first layer in ANN and SNN produces nearly the same representation. As the network goes deep, the similarity becomes lower implying SNN's latter layers extract different features from the HAR tasks as compared to ANNs. We can tentatively say that the difference in features may be the reason why SNNs and ANNs yield different accuracy on HAR tasks. We also discover that the shallow layers and the deep layers are very different, with a lower than 0.4 CKA value.

## 4. Conclusion

In this paper, we have shown the supremacy of Spiking Neural Networks (SNNs) over Artificial Neural Networks (ANNs) on HAR tasks, which, to our best knowledge, is the first. Compared to the original ANNs, SNNs utilize their LIF neurons to generate spikes through time, bringing energy efficiency as well as temporally correlated non-linearity. Our results show that SNNs achieve competitive accuracy while reducing energy significantly, and thus demonstrate the advantage of SNNs for low-power wearable devices.

## Data availability statement

The original contributions presented in the study are included in the article/supplementary material, further inquiries can be directed to the corresponding author.

## Author contributions

YL conceptualized the problem, implemented the algorithm, performed the experiments of algorithmic comparison, and wrote the initial manuscript. RY devised the hardware accelerator, performed the experiments of hardware comparison, wrote the hardware section, and gave suggestions to the manuscript. YK assisted with the experiments, gave suggestions to the manuscript, and draw several figures. PP conceptualized the problem, supervised the work, funded the project, and revised the manuscript. All authors contributed to the article and approved the submitted version.
